# The state of abortion services in five Pacific Island countries: a legislative analysis and scoping review

**DOI:** 10.1186/s12884-025-08005-0

**Published:** 2025-09-30

**Authors:** Jenny Cao, Amanda Blair, Caroline Homer, Deborah Bateson, Kirsten Black, Amanda Noovao-Hill, Leeanne Panisi, Errolyn Tungunaboe, Kamo Dumo, Liai Siitia, Alyce Wilson

**Affiliations:** 1 Women’s, Children’s and Adolescents’ Health Program, Burnet, 85 Commercial Rd, Melbourne, VIC 3004 Australia; 2 School of Population and Global Health, The University of Melbourne, 207 Bouverie St, Carlton, VIC 3053 Australia; 3 Faculty of Medicine and Health, The University of Sydney, Sydney, NSW Australia; 4 Fiji National University, Suva, Fiji; 5 National Referral Hospital, Honiara, Solomon Islands; 6 Vila Hospital, Port Vila, Vanuatu; 7 Angau Memorial General Hospital, Lae, Papua New Guinea; 8 Samoa Family Health Association, Apia, Samoa

**Keywords:** Abortion law, Unsafe abortion, Maternal mortality, Sexual and reproductive health, Health policy, Pacific Islands

## Abstract

**Background:**

Unsafe abortions are a leading cause of maternal mortality. This study aimed to conduct a legislative analysis and scoping review to i) describe the abortion laws in Papua New Guinea (PNG), Fiji, Vanuatu, Solomon Islands, and Samoa, ii) identify what safe and unsafe abortion practices and services are available in these countries, and iii) understand women's experiences of accessing these services.

**Methods:**

Abortion law data for these five countries were extracted and examined from the Global Abortion Policies Database. A scoping review identified relevant peer-reviewed and grey literature. Studies from all years and all languages were eligible. A systematic search was conducted on 1 December 2024 using Medline (Ovid), Embase (Ovid), and CINAHL (EBSCO) databases. Additionally, we hand-searched Google Scholar and the websites and databases of organisations focused on sexual and reproductive health (SRH) in the selected Pacific Island countries. Two independent reviewers screened studies for eligibility using Covidence software. An expert advisory group of Pacific Islander clinicians guided this review.

**Results:**

The legislative analysis revealed that abortion laws are generally unclear and restrictive in all five countries. Fiji has the most legal grounds for abortion (five), while Vanuatu has the fewest (one). In all five countries, abortion law specifies penalties (imprisonment) for women, providers, and anyone assisting with abortion. The scoping review included 17 articles: 10 from PNG, three from Vanuatu, one from the Solomon Islands, one from Fiji, and two from the Pacific Islands. Studies discussed various abortion strategies and experiences of post-abortion care, but none discussed safe abortion care. Misoprostol use was widely documented to induce abortion. Unsafe abortion methods included using various physical means and consuming traditional herbs. Women lacked control over abortion decision-making and described their experiences seeking post-abortion care for complications.

**Conclusion:**

We found limited evidence surrounding safe abortion services in these five countries. Future research should examine what optimal safe abortion care may look like within these countries’ health systems. Concerted advocacy is necessary to drive legislative reform, dismantle barriers, and create an enabling environment for safe abortion services, thereby facilitating the achievement of SRH and rights.

**Supplementary Information:**

The online version contains supplementary material available at 10.1186/s12884-025-08005-0.

## Key messages

**What is already known on this topic:**

Unsafe abortions are a preventable cause of maternal mortality. They occur most frequently in low- and middle-income countries where abortion laws are often restrictive and access to safe abortion services is limited.

**What this study adds:**

In this scoping review, no studies discussed safe abortion services. Studies identified focused on unsafe abortion and were predominantly based in Papua New Guinea. In the Pacific Island countries, abortion laws are unclear or restrictive and comprehensive abortion care, including safe abortion services for all legal indications and all components of post-abortion care, is limited. Consequently, women turn to unsafe abortion methods such as using physical means and consuming traditional herbs. Additionally, clandestine use of misoprostol, sometimes with inaccurate guidance, has been documented.

**How this study might affect research, practice, or policy:**

Advocacy efforts are needed to decriminalise abortion so that women can openly and safely access comprehensive abortion care.

**Supplementary Information:**

The online version contains supplementary material available at 10.1186/s12884-025-08005-0.

## Introduction

Unsafe abortions are one of the leading causes of global maternal morbidity and mortality, accounting for approximately 7·9% of maternal deaths [1]. Low- and middle-income countries (LMICs) account for approximately 88% of abortions reported globally [2]. Almost all (97%) unsafe abortions occur in LMICs, where abortion is either highly restricted by law or where safe abortion is difficult to access even when it is legally permitted [3]. Yet, unsafe abortions are one of the most preventable and neglected causes of maternal mortality [4].

The World Health Organization (WHO) defines an abortion as unsafe when it is carried out by either a person lacking the necessary skills or in an environment that does not conform to minimal medical standards, or both [5]. Abortions are safe when they are performed by adequately trained personnel using evidence-based methods appropriate to the pregnancy duration [6, 7]. Safe abortion also encompasses self-managed medical abortions for pregnancy gestational ages below 12 weeks, according to the latest WHO abortion care guidelines [7]. Full access to safe abortion care is a human right and a sexual and reproductive health (SRH) right. It is also imperative for advancing the United Nations (UN) Sustainable Development Goals (SDGs) 2030 Agenda, particularly target 3.7, which aims to ensure universal access to SRH services.

Contraceptive use in the small islands of the Pacific is lower than in other LMICs [8, 9]. Only 18–48% of all partnered women aged 15–49 years in the Pacific Islands are currently using any contraceptive method, significantly below the 62% average for low-income countries [10]. Unmet need for family planning is also high, ranging from 8 to 46% [11]. National SRH policies in the five countries studied generally support the provision of free or affordable contraception and family planning counselling [12–16]. For example, PNG’s National SRH Policy (2014) states free contraceptive access for women, girls, men, and boys in all health facilities, while Vanuatu’s Reproductive, Maternal, Newborn, Child and Adolescent Health Policy and Implementation Strategy (2017–2020) ensures everyone can access affordable family planning methods [14, 15]. However, despite these policy commitments, implementation challenges persist, and sociocultural, religious, geographical, and health service delivery-related barriers exist [17]. In particular, male involvement in reproductive decision-making, control over transport, and allocation of financial resources can influence women’s ability to access SRH services [18]. Additionally, many of the small island countries, much of the population lives in rural, often isolated areas, or atolls with limited infrastructure such as roads, electricity, and running water. As a result, health facilities are difficult to reach [17]. For those living on remote islands, geographical, logistical and economic barriers prevent them from travelling to neighbouring countries to access safe, legal abortion services [19].

There is a lack of national data on induced abortion in the Pacific Islands, and details surrounding abortion-related services are equally scarce [8]. Prevalence estimates from demographic and health surveys (DHS) suggest that between 0–4% of 20–24-year-olds have had an induced abortion [20]. However, due to the significant stigma surrounding abortion, this figure is likely to be an underestimation of the true prevalence [20]. Abortion laws in the region can be restrictive, often permitting abortion only under exceptional circumstances, such as when it is necessary to preserve a woman’s life [21]. Colonialism has profoundly shaped abortion laws and discourse in the Pacific Islands, with colonial-era legal frameworks, missionary influence, and gender norms entrenching restrictive abortion policies that persist today, reinforcing stigma and limiting reproductive autonomy [19]. Christian nationalism, views of the fetus as “life”, and portrayals of women seeking abortions as ignorant or murderous criminals shape public and political opinion and underpin abortion stigma for Pacific Island women [19]. In settings where abortion laws are restrictive, rates of unintended pregnancies ending in abortion are higher compared to those in settings with more liberal abortion laws [22]. Meanwhile, less restrictive abortion laws can reduce the rate of abortion-related morbidity and mortality [3]. While the liberalisation of abortion laws is a vital step, it may not necessarily always translate into improved access to safe abortion services [4].

Knowledge gaps in the Pacific Island countries exist regarding access to safe abortion services and post-abortion care, unsafe abortion methods, experiences of abortion care, and how these relate to the legal status of abortion in each Pacific Island country. To our knowledge, no scoping review has been conducted that explores the state of abortion services in Pacific Island countries. Therefore, this study aimed to i) describe the abortion laws in Papua New Guinea (PNG), Fiji, Vanuatu, Solomon Islands, and Samoa, ii) identify what safe and unsafe abortion practices and services are available in these countries, and iii) understand women's experiences of accessing these services.

## Methodology

### Legislative analysis

A legislative analysis was conducted to review the legislation governing abortion in PNG, Fiji, Vanuatu, Solomon Islands and Samoa. For the purposes of our study, abortion laws that are “liberal” refer to those which allow legal access to abortion under a broad set of grounds and/or gestational limits. Abortion laws are described as “defined” when they clearly specify whether abortion is allowed under different legal grounds. Data were extracted from the Global Abortion Policies Database (GAPD), an open-access database that comprehensively details the abortion laws, policies, health standards, and guidelines for the WHO and UN member states [21]. (Table 1.) Only data provided in the GAPD were extracted and examined, and there was no separate review of the individual countries’ statutes. A spreadsheet was created in Microsoft Excel, and data on whether abortion is allowed at the woman’s request, the legal grounds for abortion, penalties incurred for illegal abortions, and allowed providers were extracted. Where data were not specified according to the GAPD, this was recorded. A descriptive analysis was conducted, and the abortion legislation was mapped, described, and compared across the five selected Pacific Island countries.

### Scoping review

A scoping review was undertaken as the most suitable approach, given the need to collate and synthesise available peer-reviewed and grey literature and identify the gaps in existing literature [23]. The scoping review methodology was informed by Arksey and O’Malley’s systematic five-stage methodological framework. This involves identifying the research question, identifying relevant studies, study selection, charting the data, and collating, summarising, and reporting the results [23]. This review focuses on PNG, Fiji, Vanuatu, the Solomon Islands, and Samoa because these are the five largest and most highly populated countries in the Pacific that are also signatories of the UN SDGs for 2015- 2030.

A research protocol was developed a priori and adhered to (available upon request). An expert advisory group of leading obstetricians and gynaecologists from the five Pacific Island countries guided the research process, as well as the synthesis, interpretation, and confirmation of findings.

The Population, Concept, and Context framework, recommended by the Joanna Briggs Institute for scoping reviews, underpinned the development of the search terms and inclusion and exclusion criteria [24]. The population included women with experiences of abortion; the concepts included safe abortion services, unsafe abortion methods, and post-abortion care; and the context included PNG, Fiji, Vanuatu, the Solomon Islands and Samoa. Abortion is considered safe when performed or supported by a trained person using WHO-recommended methods appropriate for the pregnancy duration. The latest WHO abortion care guidelines also recognise the self-management of medical abortion (using mifepristone plus misoprostol or misoprostol alone) at gestational ages less than 12 weeks as safe [7]. Meanwhile, unsafe abortion is defined as when a pregnancy is terminated either by persons lacking the necessary skills or in an environment that does not conform to minimal medical standards or both [7]. Multiple study designs were eligible for inclusion, including qualitative studies, quantitative studies, case–control studies, cross-sectional studies, retrospective or prospective cohort studies, case series, and program/service evaluations. Grey literature reports were also eligible for inclusion. Previously conducted reviews were excluded. Editorials, commentaries, and opinion pieces were also excluded, as they do not follow a research methodology.

#### Search strategy and selection criteria

The search strategy, including the search terms (Supplementary Table 1), was used to identify studies that addressed the research question and met the inclusion criteria. Studies in all languages and from all years were eligible. The databases MEDLINE (Ovid), Embase (Ovid), and CINAHL (EBSCO) were accessed. Google Scholar, websites, and databases of non-governmental organisations (NGOs), and organisations that focus on women’s SRH and operate in the five selected Pacific Island countries were searched for relevant studies. This included the United Nations Population Fund, WHO, Population Services International, countries’ Ministries of Health, International Women’s Development Agency, International Planned Parenthood Federation, Pacific Society for Reproductive Health, and MSI Reproductive Choices.

The search was conducted on 1 December 2024 and returned 4020 studies, with a total of 2008 duplicates removed. The titles and abstracts of 2012 studies were independently screened, using Covidence software by two reviewers (JC and AB). Discrepancies were discussed between the two reviewers to reach a consensus and any disagreements were resolved through consultation with a third reviewer (AW). 1941 studies were excluded at the title and abstract stage. 64 studies’ full texts were reviewed, and 50 studies were excluded, with reasons for exclusion reported. All studies’ reference lists were screened for eligible studies and one eligible report was identified.

**Fig. 1:**
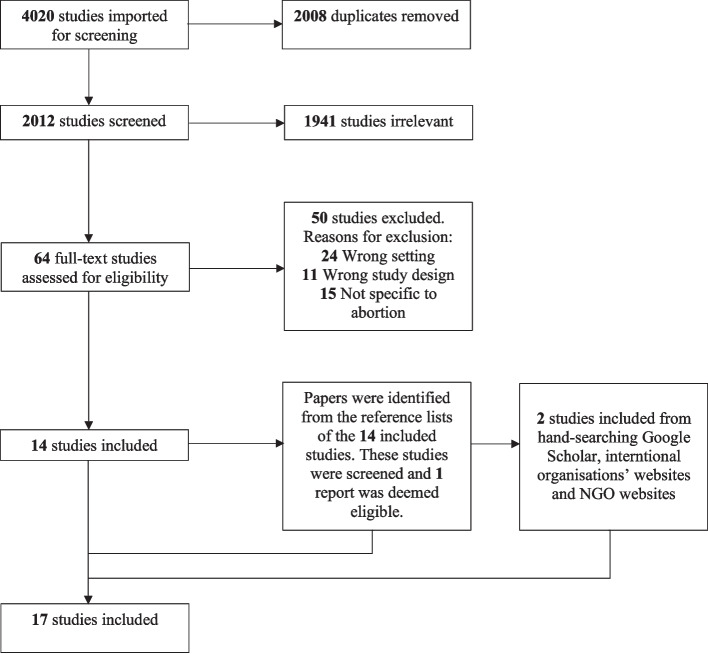
PRISMA flow diagram

Further hand-searching of Google Scholar, international organisations’ and relevant NGOs’ websites was conducted to identify additional relevant peer-reviewed and grey literature not captured through the database search. This process identified two additional relevant papers. Ultimately, 17 studies were included in the review (Fig. 1).

A data extraction form was created using Microsoft Excel and key information was charted including author/s, year of publication, study country/setting; aim, relevance to safe abortion, unsafe abortion, or post-abortion care, study design, methods, and sample size. Key findings relevant to safe abortion, unsafe abortion, and post-abortion care, according to the research questions were also extracted.

Participant study quotes and case studies were charted as well as study strengths and limitations. All extracted data was reviewed by the second reviewer, AB, to ensure the data-extraction process was robust. A descriptive analysis of findings was conducted as only five of the included studies were qualitative.

### Patient and public involvement

Patients or members of the public were not directly involved in this research study. Advisory group members were from authors’ professional SRH networks in the Pacific Islands and they were each invited to collaborate on this study.

These research findings will be shared widely with local community organisations, international NGOs, research institutes and medical practitioners in the five Pacific Island countries through authors’ professional networks and associations. In addition, these research findings have been presented at relevant SRH conferences such as Pacific Society for Reproductive Health, International Federation of Gynaecology and Obstetrics, and Perinatal Society of Australia and New Zealand.

## Results

### Legislative analysis

The legislation related to induced abortion was examined for PNG, Fiji, Vanuatu, Solomon Islands, and Samoa (Table 1).

Fiji was found to have the greatest number of legal grounds for abortion in its legislation. Abortion is permitted in cases of fetal impairment, rape, or incest, to preserve a woman’s mental or physical health, or save her life. Legal grounds are not specified around whether abortion is permitted for economic or social reasons, if the woman has an intellectual or cognitive disability, or for preserving the health of the woman.

Abortion to save a woman’s life is permitted in the legislation of four out of the five countries reviewed, that is, Fiji, PNG, Samoa, and Solomon Islands. In Vanuatu, the legislation does not specify whether abortion to save the life of the woman is legal. Although the legal grounds for abortion are not specified in Vanuatu, the penal code includes a broad statement that abortion would be legal “if the miscarriage procured constituted a termination of pregnancy for good medical reasons.” There is no clear definition for “good” medical reasons.

In Fiji, PNG and Solomon Islands the legality of abortion at the woman’s request (without identifying any reasons e.g. health, rape, incest etc.) is not specified in the countries’ penal or criminal code. It is unclear if a woman requesting an abortion could be charged with an offence. In Samoa and Vanuatu, the penal code states that abortion at the woman’s request is illegal. Of the five countries examined, Samoa’s Crimes Act 2013 is the clearest and outlines all legal grounds for abortion. This includes abortion in cases where it is necessary to preserve the mental health, physical health, or life of the woman. In all other cases, abortion is illegal.

In all five countries, there are penalties for unlawful/illegal abortions. In addition, any provider who assists the woman in procuring an abortion can face imprisonment, although the severity of penalties varies between countries. The most severe penalties for providers exist in the Solomon Islands, where they may face life imprisonment. Any person who assists the woman in procuring an abortion is subject to the most severe penalties in Samoa, with up to seven years imprisonment. In all five countries, penalties for the woman having an abortion also apply, the Solomon Islands has the most severe punishment being life imprisonment.

Only Fiji specifies who can provide abortion services. In Fiji, a medical practitioner defined in the Crimes Decree 2009 as “any person lawfully registered under a law of Fiji to practice as a medical practitioner” can provide abortion services. None of the legislation for the five countries reviewed specifies where abortion or post-abortion services can be provided [21].

**Table 1:** Abortion at the woman’s request, legal grounds for abortion and penalties incurred for illegal abortions in the Pacific Island countries

Pacific Island countries						
		**Fiji**	**Papua New Guinea**	**Samoa**	**Solomon Islands**	**Vanuatu**
**Abortion at the woman's request**		NS	NS	N	NS	N
**Legal Grounds**	***Economic or Social Reasons***	NS	NS	N	NS	NS
	***Foetal Impairment***	Y	NS	N	NS	NS
	***Rape***	Y	NS	N	NS	NS
	***Incest***	Y	NS	N	NS	NS
	***Intellectual or cognitive disability of the woman***	NS	NS	N	NS	NS
	***Mental Health***	Y	NS	Y	NS	NS
	***Physical Health***	Y	NS	Y	NS	NS
	***Health***	NS	NS	N	NS	NS
	***Life***	Y	Y	Y	Y	NS
	***Other***	NA	Y	NA	Y	Y
**Penalties**	***For provider***	Y	Y	Y	Y	Y
	***For a person who assists***	Y	Y	Y	Y	Y
	***For the woman***	Y	Y	Y	Y	Y
**Years of imprisonment**	***For provider***	14 years	14 years	7 years	life	2 years
	***For a person who assists***	3 years	3 years	7 years	5 years	2 years
	***For the woman***	7 years	7 years	7 years	life	2 years

NS (not specified), N (No), Y (Yes)

### Scoping review

Ultimately, 17 articles were included in the scoping review. Of these, 14 were journal articles and three were reports, 10 were from PNG, three from Vanuatu, one from the Solomon Islands and one from Fiji. No articles were identified from Samoa. Two articles focused on the Pacific Islands more broadly. This included all five of our countries of focus. Five papers were qualitative studies, involving analysis of data from individual interviews, focus groups, court documents or articles, opinion pieces and letters to the editor, eight were quantitative studies, involving medical record reviews and survey data, and four studies employed mixed-methods. Articles were published between 1974 to 2024. Topics covered by papers included unsafe abortion (*n* = 15), post-abortion care (*n* = 7), and private abortion care (*n* = 2). (Supplementary Table 2.)

#### Abortion and post-abortion care services and practices

##### Safe abortion

Limited studies were identified that discussed safe abortion services or models of safe abortion care in the five Pacific Island countries. Vallely et al.’s study in PNG, based at The Eastern Highlands Provincial Hospital in Goroka, reported that no safe abortion services were delivered by their hospital during the six-month study period. [25] They reviewed case records of women presenting with abortion complications over the same period. It was found that out of the 14 identified women using misoprostol, the two who received a dose of misoprostol appropriate for their gestation received the misoprostol from hospital-based healthcare workers (HCWs) [25]. Both women were in their second trimester. Another PNG study surveyed women who had exchanged sex for money or goods and undergone an abortion for unintended pregnancies [26]. It found that 17% (*n* = 23) had undergone a termination by a medical professional and that in 5% of cases, the method of abortion was “medical surgery” [26]. However, it was unclear whether it was medical, surgical or safe according to WHO definitions [7].

##### Misoprostol

Misoprostol was a commonly reported abortion strategy to manage unintended pregnancies. Three studies from PNG reported the prevalence of different abortion methods used (Table 2). Asa et al. [27] and Vallely et al. [28] both found that misoprostol was the most frequently used abortion method, reported by 85% and 39% of their participants, respectively. In Vallely et al.’s [28] study, women described using two to five tablets used through oral and vaginal routes. Misoprostol was reported to be easily accessible by 75% (n = 51) of women interviewed who had a misoprostol-induced abortion [28]. The scoping review advisory group also confirmed the widespread informal use of misoprostol for abortions across the five Pacific Island countries.

Despite its widespread use, challenges remain in ensuring the correct dosage and timing. Two studies highlighted that women who sourced their misoprostol from outside the health services were sometimes provided with inaccurate instructions, dosage, and advice for their gestation [25, 28]. In a 2014 study, Vallely et al. [25] found that 86% (n = 14) of study participants using misoprostol for abortions were administering incorrect dosages. However, in 2021, more recent evidence from Bolnga et. al’s [29] prospective observational study revealed that 35% (n = 51) of participants who used misoprostol for abortion used incorrect dosages.

**Table 2:** Different abortion methods reported

Study	Country	Sample size	Misoprostol	Traditional Herbs	Physical means	Other
Asa et al. 2012 [27]	PNG	*n* = 27	85% (23/27)	7% (2/27)	7% (2/27)	N/A
USAID 2011 [26]	PNG	NS	23%^a^	45%	9%^b^	18%
Vallely et al. 2014 [25]	PNG	*n* = 28	39% (11/28)	11% (3/28)	21% (6/28)	11% (3/28)

^a^ This study mentioned using tablets and did not specify misoprostol

^b^ Massage was the physical means of abortion measured in this study

*N/A* Not applicable

*NS* Not specified

##### Unsafe abortion

Most forms of abortion identified in this review were unsafe. In USAID’s [26] study in PNG, 23% of women reported using ‘tablets’, although what these tablets were was not described.

In PNG, women described using physical means to terminate their pregnancy including squeezing or tying a rope around the abdomen, excessive exercise, running over mountains, jumping over streams, falling onto their abdomen, hard physical work, and insertion of a stick into the vagina [28]. Physical means of inducing abortion were also reported in the Solomon Islands and Vanuatu such as massage, climbing trees, extended exercise (running, jumping and skipping), lifting heavy leads while working, falling or jumping down from trees or hitting, kneeing, jumping, or walking on the stomach or back while lying on the ground [30, 31]. Traditional herbs were widely reported to be used to induce abortion in PNG, Solomon Islands and Vanuatu [25–28, 30–33]. This included tree bark or grasses chewed and swallowed or squeezed to make juice, and other concoctions with leaves, flowers, ginger, lime, or unripe papaya juice that were consumed [28, 31]. One PNG-based study analysed the causes of hospital and health centre maternal deaths between 1971 and 1972, finding that four out of 364 deaths were attributed to “criminally induced” abortions [34].

##### Post-abortion care

Most studies that referenced post-abortion care (spontaneous and induced) were based in PNG and focused on hospital-based care. Varied management of post-abortion complications was described, including evacuation or dilatation and curettage for retained products and antibiotics [25, 27]. Some women presented to the hospital seeking to be “cleaned” to ensure the remaining conception products were cleared [28]. In 2014, the Vanuatu Family Health Association clinics reported providing 390 post-abortion counselling services [30]. In the Solomon Islands, post-abortion care providers included nurses and doctors in public facilities, and pharmacists, doctors and traditional doctors in private settings [31].

#### Women’s experiences of abortion and post-abortion care

##### Decision-making

In PNG and Fiji, studies highlighted women’s lack of control over decision-making around abortions. Women described instances of sexual and reproductive coercion, and many felt pressured by their partners to have an abortion when their partners did not want the baby [28, 35]. Boyfriends, husbands, and family members were also often involved in the decision-making process and determining the means for the abortion [28, 35]. According to two women in PNG:

*“……he said to me,"I don’t want you to do that (be pregnant), I have a lot of friends so I will get this Cytotec and come and give you and you will end this pregnancy” …. I was happy that he came and gave it to me, and I ended this pregnancy….”*Noreen, 20 years, grade 8 student (PNG) [28].

*“My husband brought me to see a relative at the hospital….he did not want the baby, so he brought me….[to get an abortion]”.*Mary, 30–34 years, housewife (PNG) [28].

In Burry et al.’s [36] qualitative analysis of 18 court cases of illegal abortions in the Pacific Islands, five young women experienced reproductive coercion from powerful individuals. Among them, two 14-year-old girls became pregnant after being sexually abused by older men. One girl, who was sexually abused by her uncle, was coerced by her aunt into undergoing an abortion without her full informed consent or understanding of the procedure. Another girl was sexually abused by a man paying for her education with financial control over her. She did not report the abuse due to his threats of physical violence. To induce an abortion, he fed her strong tea, raw eggs, and rum mixed with milk [36].

In PNG, Agyemang-Duah et al. [37] showed that women who had experienced intimate partner violence (IPV) in the last 12 months before the PNG DHS 2016–2018 survey were significantly more likely to terminate a pregnancy compared to those who did not experience IPV. Maviso et. al [38] corroborated these results. These two studies posited that in abusive relationships, the husband or partner may not want the baby and may use violence or other coercive means to force the woman to have an abortion [37, 38]. Additionally, many women expressed fear of raising a baby without the support of their partner [28, 33, 35]. One Fiji-based study included a case study of a young woman being pressured by her partner to have an abortion at a private clinic, although it is unclear whether this would be classified as a safe abortion per WHO’s definition [5, 35].

*"Rachel resisted, but over the next 2 weeks, Mikaele continued to pressure her to have an abortion. He went as far as obtaining the details of a doctor and organizing an appointment for Rachel, and later insisting she go through with the procedure. Feeling overwhelmed and unsure whether she could raise a baby without the support of Mikaele, she yielded to his pressure and agreed to the abortion."*Case study (Fiji) [35].

In the Solomon Islands, some women autonomously made their own decisions about whether to undergo abortion, whilst other women took advice from their partners, friends, mothers, other female relatives or HCWs [31]. A woman was less likely to confide in her father or other male relatives due to fear of verbal or physical violence [31].

Women described several reasons for desiring an abortion, including individual socioeconomic circumstances, medical issues, relationship problems, sociocultural factors, and stigma associated with having a child outside of marriage [28–31, 35]. Younger and single women expressed feeling ‘not ready’ for a baby, concerned it would interrupt their education [28]. Fear of reactions from parents, partners, family members, and community members was also a key motivator for women seeking abortions [36]. Notably, in PNG, women who were aware of modern contraceptive methods and made independent decisions about contraceptive use were 3.38 and 2.54 (adjusted Odds Ratio = 3.38; 95% Confidence Interval: 1.39–8.18 and adjusted Odds Ratio = 2.54; 95% Confidence Interval: 1.18–5.45, respectively) more likely to undergo abortions [38].

Women often delay seeking post-abortion care by up to four weeks to avoid disclosure about having an abortion [28]. Signs and symptoms, such as feeling dizzy, back pain, abdominal pain, blood clotting or prolonged/excessive vaginal bleeding were common prompts for women to seek care [28]. Stigma and fears of prosecution were reported as reasons for women not disclosing that they had an induced abortion, often only discovered upon clinical observation [28]. Instances of women seeking post-abortion care too late were described in the literature, where women were dead upon arrival at the emergency department [27].

##### Cost

In PNG, women reported that misoprostol was expensive and described paying K200 (USD $56·90) for two tablets from HCWs, and paying K40 (USD $11·38) and a traditional basket of high value [28]. These were described as very high payments by women in this study. In PNG, one woman reported obtaining traditional herbs from a “woman in the village” for K20 (USD $5·69), while another obtained “ginger” from a “medicine man” costing her K10 (USD $2·84) [28]. In another study, 36.4% (n = 51) of women reported the cost of misoprostol to be over K587 (USD $165·81) [29]. In the Solomon Islands, traditional doctors were reported to provide their services in exchange for payments of SBD 100–700 (USD $28·25—$197·73), food, betel nut, cigarettes, or sex [31].

##### People who assisted

Misoprostol was obtained by women with the help of friends or relatives, from private pharmacies (over the counter or with prescriptions from HCWs), or directly from doctors or hospitals [28, 31, 39]. HCWs also assisted women with administration [27]. One case in the Pacific Islands identified a nurse who admitted to performing abortions on 20 to 30 women at their request [36]. She was charged with 16 separate counts of providing surgical abortions for women at the hospital, their homes, public housing, or motels [36]. However, it was not specified which Pacific Island country this case occurred in. Asa et al. [27], Bolnga et al. [29] and USAID’s [26] studies quantitatively reported different people who assisted women with their abortions (Table 3). These people included HCWs, friends, relatives, neighbours, and traditional healers. In PNG, USAID found that 83% (*n* = 23) of women reported that their last abortion was conducted without HCWs’ assistance, highlighting the extent to which abortion occurs outside of formal medical settings [26]. Furthermore, Burry et al. ‘s [36] qualitative court data analysis identified seven HCWs and five untrained abortion providers charged with illegal abortions in Pacific Island countries between 1967 and 2014. In Vanuatu and PNG, some young women described accessing traditional healers for abortion [40].

**Table 3:** Who assisted the women with their abortions?

Study	Country	Sample size	Health Care Workers	Friend(s) or relative(s)	Friend(s) or neighbour(s)	Traditional Healer	Self-administered
Asa et al. [27]	PNG	*n* = 27	52% (14/27)	22% (6/27)	N/A	N/A	N/A
Bolnga et al. [29]	PNG	*n* = 51	63% (32/51)	37% (19/51)	N/A	N/A	N/A
USAID [26]	PNG	*n* = 23	17% (4/23)	N/A	48% (11/23)	13% (3/23)	22% (5/23)

Percentages (%) represent the proportion of women reporting assistance from each type of provider or individual

*N/A* – Not applicable

## Discussion

This scoping review examined the state of abortion services including, safe and unsafe abortion, post-abortion care and women’s experiences of abortion in PNG, Fiji, Vanuatu, Solomon Islands and Samoa. A legislative analysis of abortion laws in these countries was also conducted. Despite a rigorous literature search, documentation of safe abortion services was not identified. This absence reflects HCWs’ inability to provide safe abortion services due to the restrictive nature of abortion laws in these five countries where abortion is only allowed under certain legal grounds. In addition to legal restrictions, women experience challenges in accessing safe abortion services due to costs, lack of quality services, and stigma. We identified a variety of unsafe abortion methods used including physical means and traditional herbs, consistent with findings from other LMICs [41–43]. These challenges highlight the urgent need for increased access to safe abortion services for women in cases where abortion is legal [44].

This study found that most of the limited research available was based in PNG. Further research across the Pacific Islands is required to better understand women’s access to and experiences of safe abortion services and post-abortion care. In addition to exploring women’s experiences, HCWs’ attitudes to abortion and experiences of providing abortion should also be investigated. HCWs play a pivotal role in providing comprehensive abortion care [45, 46]. As such, their attitudes and personal views towards abortion can facilitate and obstruct service provision [45, 46]. Generating evidence on these issues is needed to advocate for legal reform and further investment into increasing access to safe abortion services.

Our findings indicate that misoprostol is widely being used for abortion outside of formal health services. Misoprostol offers a safer and effective option for abortion and is associated with less maternal morbidity and mortality [47–50]. It is used for various medical purposes and is therefore readily available [51, 52]. We found that access to misoprostol often occurs through informal networks, including HCWs and pharmacists, aligning with findings from other multi-country surveys [53]. Some HCWs were reported to assist women seeking abortion by illegally supplying or administering misoprostol. However, some women who used misoprostol received inaccurate advice regarding correct dosage and administration, highlighting the need to improve the promotion of accurate information on its correct use. The WHO abortion care guidelines emphasise that in contexts where abortion access is restricted, ensuring that women have access to precise and adequate information on misoprostol use outside of medical settings is essential [7]. Even in restrictive legal environments, the use of misoprostol is likely to persist, making accurate guidance crucial. Despite the restrictive abortion laws in the Pacific Island countries we examined, post-abortion care provision is accepted as part of standard women’s health care, and women experiencing complications from unsafe abortion need access to care [54]. Hence, access to high-quality, comprehensive post-abortion care must be strengthened to include immediate treatment of complications, contraceptive counselling and service provision, care for sexually transmitted infections and HIV, and community empowerment through awareness and mobilisation [55]. Before abortion was legalised in Uruguay, one major risk and harm reduction strategy focused on providing information about various abortion methods, including evidence-based information about misoprostol (e.g. dose, routes, symptoms, side effects, mechanisms of action, effectiveness, Moebius syndrome, problems of use at late gestational ages that might cause premature birth). Post-abortion care was also offered as part of this approach [56]. This model effectively reduced maternal morbidity and mortality in Uruguay [57]. Adopting similar approaches in the Pacific Island countries could help improve the safety of abortions.

We found that men had significant control over women’s decision-making around abortion. Studies from other LMICs have also found that men often have control over women’s SRH choices, including decisions about abortions [58–61]. Our review found that men, particularly partners, often took the lead in navigating abortion services and arranging for abortions to take place without the woman’s consent. These power imbalances exist within a socio-cultural context steeped in gender inequity and are exacerbated by economic and educational inequities, which limit women’s SRH autonomy [62]. Although there was a lack of studies on safe abortion, we found examples of women being pressured by a partner to undertake an abortion. This speaks to whether the WHO definition needs to be expanded to include that the person getting the abortion is fully informed and is consenting without coercion or pressure from others [7]. Beyond direct coercion, women may also be motivated to have an abortion due to fear of their partner not supporting the baby, a finding echoed by Rehnstrom et al.’s [61] study in Kenya. Additionally, women’s relationship with their partner could also influence their decision to seek an abortion as has been previously documented [58, 63]. The significant influence of men on women’s abortion-related care can undermine women’s autonomy, impeding universal SRH rights [58].

Women may be deterred from seeking an abortion or post-abortion care due to fear of legal repercussions, stigma, and poor treatment from HCWs [64]. None of the laws in the five countries we reviewed explicitly specify whether abortion at a woman’s request is legal. Of these countries, Fiji has the most liberal abortion laws, while Samoa’s laws are the most defined. However, the abortion laws of these five countries are generally restrictive and unclear. This lack of clarity is common in LMICs, where there is often an incongruity between the abortion law and its implementation in practice [65]. Restrictive abortion laws disproportionately impact LMICs, increasing the prevalence of unsafe abortion and resulting in high maternal mortality rates [66].

In the Pacific Island countries, there is an urgent need for the decriminalisation of abortion to enable skilled practitioners to provide abortion services openly and safely [4, 67]. International organisations increasingly regard the denial of safe abortion services as a human rights violation [4], and the UN has urged its member states to decriminalise abortion [67]. All five of the selected Pacific Island countries have ratified the Convention on the Elimination of All Forms of Discrimination against Women (CEDAW) [21]. Yet, despite this commitment, they have failed to bring their domestic laws in line with CEDAW’s standards. The CEDAW Committee has raised concerns that the restrictive abortion laws in PNG, Fiji, Solomon Islands and Samoa undermine women's rights to life, health, and personal security [19, 68–71]. However, liberal abortion laws alone are insufficient to guarantee access to safe abortion services. For instance, despite the legalisation of abortion in India and Zambia in the 1970 s, unsafe illegal abortion persists due to the unavailability of safe, legal abortion services and over-burdened and under-resourced healthcare systems [4, 72]. The connection between abortion law, policy, and service access is complex and interconnected to the socioeconomic, cultural and political context [73]. Women’s limited knowledge of the abortion law, lack of provisions for providers on how to interpret them, and socio-cultural barriers such as religion and moral beliefs, all interplay to encumber women’s access to safe abortion services [4, 66, 74].

Strengths of this study include the use of a broad search strategy to ensure all relevant research was captured. An expert advisory group of leading senior obstetricians and gynaecologists from each of the five Pacific Island countries provided guidance and oversight. Limitations include a focus on five of the most highly populated and largest countries in the Pacific, despite the Pacific comprising over 25 nations and territories. Limiting the scope of this review to five countries was a pragmatic decision to ensure that the review could be conducted within the available time and resources, while still capturing the variation in the region. Whilst we searched the grey literature for relevant reports, we were limited by search engine capability and publicly available documents reviewed. Although our scoping review included studies in all languages in its eligibility criteria, we were unable to identify studies in languages other than English, which may result in an underrepresentation of local and community perspectives. Additionally, there is limited literature from the Pacific Islands that describes the impact of legislation on abortion care. For example, Burry et al. [36] analysed 18 court cases from 12 Pacific Island countries. We included this study because the five Pacific Island countries in our review were among those listed. However, it remains unclear which specific countries the court cases discussed originated from, and whether any pertain to the five countries included in our review. Our legislative analysis was limited to data provided by the GAPD and did not involve a review of each country’s statutes. We also did not examine legislation related to conscientious objection, requirements of parental consent for minors, and substitute decision-making for those with intellectual disability [21]. Moreover, the legislative analysis does not capture the complexity of what occurs on the ground in the Pacific with regard to the varying interpretations of the law by the community, service providers, and law enforcement officers. To this effect, the advisory group reported that what is written in the law may not be enforced in actual practice.

## Conclusion

We examined abortion services and laws in five LMICs in the Pacific. We found a lack of evidence regarding safe abortion care, largely reflective of the restrictive abortion laws. Misoprostol was commonly used for abortion, but unsafe methods, including various physical means and traditional herbs, were also reported. Decriminalising abortion is necessary to improve SRH outcomes. Women need greater access to modern contraception and safe abortion care by appropriately trained and skilled providers within an enabling environment. Community-driven advocacy and programs are vital to shifting rigid gender and sociocultural norms regarding contraception use and abortion access, ultimately empowering women to have more control over their own SRH

## Supplementary Information

Supplementary Material 1

Supplementary Material 2

## Data Availability

The datasets used and/or analysed during the current study available from the corresponding author on reasonable request. All data generated or analysed during this study are included in this published article [and its Supplementary information files].

## Abbreviations

CEDAW: Convention on the Elimination of All Forms of Discrimination against Women
DHS: Demographic and Health Survey
GAPD: Global Abortion Policies Database (s)
HCWs: Healthcare workers
IPV: Intimate partner violence
LMICs: Low- and middle-income countries
PNG: Papua New Guinea
SDGs: Sustainable Development Goals
SRH: Sexual and reproductive health
UN: United Nations
WHO: World Health Organization

## References

[1] Say L, Chou D, Gemmill A, Tunçalp Ö, Moller AB, Daniels J, et al. Global causes of maternal death: a WHO systematic analysis. Lancet Glob Health. 2014;2(6):e323–33. 25103301 10.1016/S2214-109X(14)70227-X

[2] Chae S, Desai S, Crowell M, Sedgh G, Singh S. Characteristics of women obtaining induced abortions in selected low- and middle-income countries. Gebhardt S, editor. PLoS ONE. 2017;12(3):e0172976. 10.1371/journal.pone.0172976 PMC5371299 28355285

[3] Haddad LB, Nour NM. Unsafe abortion: unnecessary maternal mortality. Rev Obstet Gynecol. 2009;2(2):122–6. 19609407 PMC2709326

[4] Grimes DA, Benson J, Singh S, Romero M, Ganatra B, Okonofua FE, et al. Unsafe abortion: the preventable pandemic. Lancet. 2006;368(9550):1908–19. 17126724 10.1016/S0140-6736(06)69481-6

[5] World Health Organization. The Prevention and management of unsafe abortion: report of a technical working group. Geneva. 1992;12–15:1993.

[6] Ganatra B, Tunçalp Ö, Johnston HB, Johnson BR Jr, Gülmezoglu AM, Temmerman M. From concept to measurement: operationalizing WHO’s definition of unsafe abortion. Bull World Health Organ. 2014;92(3):155–155. 24700971 10.2471/BLT.14.136333 PMC3949603

[7] World Health Organization. Abortion care guideline. Geneva: World Health Organization; 2022. Available from: https://iris.who.int/handle/10665/349316.

[8] Dawson A, Ekeroma A, Wilson D, Noovao-Hill A, Panisi L, Takala B, et al. How do Pacific Island countries add up on contraception, abortion and reproductive coercion? Guidance from the Guttmacher report on investing in sexual and reproductive health. Reprod Health. 2021;18(1): 68. 33766064 10.1186/s12978-021-01122-x PMC7992794

[9] United Nations Economic and Social Council. Advancing gender equality and universal access to sexual and reproductive health and reproductive rights. Bangkok: United Nations Economic and Social Council; 2018. Available from: https://digitallibrary.un.org/record/3882033?ln=en&v=pdf.

[10] United Nations Population Fund. Population and development profiles: Pacific Island countries. Suva, Fiji: United Nations Population Fund, Pacific Sub-Regional Office; 2014. Available from: https://pacific.unfpa.org/sites/default/files/pub-pdf/web__140414_UNFPAPopulationandDevelopmentProfiles-PacificSub-RegionExtendedv1LRv2_0.pdf.

[11] United Nations Department of Economic and Social Affairs. World Family Planning 2017 Highlights. New York: United Nations; 2017.

[12] Solomon Islands Government, Ministry of Development Planning and Aid Coordination. National Population Policy 2017–2026. Honiara: Ministry of Development Planning and Aid Coordination; 2016.

[13] Ministry of Health. National Sexual Reproductive Health Policy 2018–2023 [Internet]. Samoa; [cited 2025 Mar 1]. Available from: https://www.undp.org/sites/g/files/zskgke326/files/migration/pacific/Vanuatu-Reproductive-Maternal-Newborn-Child-Adolescent-Health-Policy.pdf

[14] National Department of Health. National Sexual Reproductive Policy [Internet]. Papua New Guinea; 2014 [cited 2025 Mar 1]. Available from: https://www.health.gov.pg/pdf/NSRPolicy_2016.pdf

[15] Ministry of Health. Vanuatu Reproductive, Maternal, Newborn, Child and Adolescent Health Policy and Implementation Strategy 2017–2020 [Internet]. Vanuatu; [cited 2025 Mar 1]. Available from: https://www.undp.org/sites/g/files/zskgke326/files/migration/pacific/Vanuatu-Reproductive-Maternal-Newborn-Child-Adolescent-Health-Policy.pdf

[16] Ministry of Health. Reproductive Health Policy [Internet]. Fiji; 2014 [cited 2025 Mar 1]. Available from: https://www.health.gov.fj/wp-content/uploads/2014/09/1_Reproductive-Health-Policy.pdf

[17] Harrington RB, Harvey N, Larkins S, Redman-MacLaren M. Family planning in Pacific Island Countries and Territories (PICTs): a scoping review. PLoS ONE. 2021;16(8): e0255080. 34351949 10.1371/journal.pone.0255080 PMC8341522

[18] Davis J, Vyankandondera J, Luchters S, Simon D, Holmes W. Male involvement in reproductive, maternal and child health: a qualitative study of policymaker and practitioner perspectives in the Pacific. Reprod Health. 2016;13(1):81. 10.1186/s12978-016-0184-2 PMC4947267 27423461

[19] Burry K, Beek K, Worth H, Vallely L, Haire B. Framings of abortion in Pacific Island print media: qualitative analysis of articles, opinion pieces, and letters to the editor. Sexual and Reproductive Health Matters. 2023;31(1):2228113. 37436430 10.1080/26410397.2023.2228113 PMC10339763

[20] United Nations Population Fund, United Nations Educational, Scientific and Cultural Organization, World Health Organization. Sexual and reproductive health of young people in Asia and the Pacific: a review of issues, policies and programmes. Bangkok: United Nations Population Fund, Asia and the Pacific Regional Office; 2015. Available from: https://asiapacific.unfpa.org/en/publications/sexual-and-reproductive-health-young-people-asia-and-pacific.

[21] World Health Organization. Global Abortion Policies Database [Internet]. 2017. Available from: https://abortion-policies.srhr.org

[22] Bearak J, Popinchalk A, Ganatra B, Moller AB, Tunçalp Ö, Beavin C, et al. Unintended pregnancy and abortion by income, region, and the legal status of abortion: estimates from a comprehensive model for 1990–2019. Lancet Glob Health. 2020;8(9):e1152–61. 32710833 10.1016/S2214-109X(20)30315-6

[23] Arksey H, O’Malley L. Scoping studies: towards a methodological framework. Int J Soc Res Methodol. 2005;8(1):19–32.

[24] Aromataris E, Munn Z, editors. JBI manual for evidence synthesis. Adelaide: JBI; 2020. Available from: https://synthesismanual.jbi.global.

[25] Vallely LM, Homiehombo P, Kelly-Hanku A, Kumbia A, Mola GDL, Whittaker A. Hospital Admission following Induced Abortion in Eastern Highlands Province, Papua New Guinea – A Descriptive Study. Vitzthum VJ, editor. PLoS ONE. 2014;9(10):e110791. 10.1371/journal.pone.0110791 PMC4201559 25329982

[26] USAID. Behaviours Knowledge Exposure to Interventions Report from a behavioural surveillance survey. Port Moresby, Papua New Guinea; 2011.

[27] Asa I, De Costa C, Mola G. A prospective survey of cases of complications of induced abortion presenting to Goroka hospital, Papua New Guinea, 2011. Aust N Z J Obstet Gynaecol. 2012;52(5):491–3. 22694099 10.1111/j.1479-828X.2012.01452.x

[28] Vallely LM, Homiehombo P, Kelly-Hanku A, Whittaker A. Unsafe abortion requiring hospital admission in the Eastern Highlands of Papua New Guinea - a descriptive study of women’s and health care workers’ experiences. Reprod Health. 2015;12(1):22. 25889957 10.1186/s12978-015-0015-x PMC4409713

[29] Bolnga JW, Lufele E, Teno M, Agua V, Ao P, Dl Mola G, et al. Incidence of self-induced abortion with misoprostol, admitted to a provincial hospital in Papua New Guinea: a prospective observational study. Aust N Z J Obstet Gynaecol. 2021;61(6):955–60. 34350583 10.1111/ajo.13413

[30] Vanuatu Family Health Association. The context of induced abortion in Vanuatu: operation research report. Port Vila: Vanuatu Family Health Association; 2014.

[31] Ministry of Health and Medical Services; Solomon Islands Planned Parenthood Association; Monash University; Burnet Institute. Addressing preventable maternal mortality and morbidity: the determinants and consequences of unsafe abortion in Solomon Islands: initial report. Honiara: [joint publishers]; 2015.

[32] Bourdy G, Walter A. Maternity and medicinal plants in Vanuatu I. The cycle of reproduction. Journal of Ethnopharmacology. 1992;37(3):179–96. 10.1016/0378-8741(92)90033-n 1453707

[33] Sanga K, De Costa C, Mola G. A review of maternal deaths at Goroka General Hospital, Papua New Guinea 2005–2008. Aust N Z J Obstet Gynaecol. 2010;50(1):21–4. 20218992 10.1111/j.1479-828X.2009.01116.x

[34] Babona G, Bird GC, Johnson DG. Maternal mortality in Papua New Guinea 1971 and 1972. P N G Med J. 1974;17(4):331–4. 4534252

[35] Mitchell E, Bennett LR. Pressure and Persuasion: Young Fijian Women’s Experiences of Sexual and Reproductive Coercion in Romantic Relationships. Violence Against Women. 2020;26(12–13):1555–73. 31663433 10.1177/1077801219882505

[36] Burry K, Beek K, Vallely L, Worth H, Haire B. Illegal abortion and reproductive injustice in the Pacific Islands: A qualitative analysis of court data. Dev World Bioeth. 2023;23(2):166–75. 35467067 10.1111/dewb.12352

[37] Agyemang-Duah W, Asare BYA, Adu C, Agyekum AK, Peprah P. Intimate partner violence as a determinant of pregnancy termination among women in unions: evidence from the 2016–2018 Papua New Guinea Demographic and Health Survey. J Biosoc Sci. 2024;56(1):141–54. 37211884 10.1017/S002193202300007X

[38] Maviso M, Aines PZ, Potjepat G, Geregl N, Mola G, Bolnga JW. Prevalence of pregnancy termination and associated factors among married women in Papua New Guinea: A nationally representative cross-sectional survey. Buh A, editor. PLoS ONE. 2024;19(9):e0309913. 10.1371/journal.pone.0309913 PMC11376535 39236064

[39] Kopunye F, Mola G, Woods C, De Costa C. Induced abortion in Papua-New Guinea: experience and opinions of health professionals. Aust N Z J Obstet Gynaecol. 2021;61(6):961–8. 34585744 10.1111/ajo.13433

[40] Kennedy EC, Bulu S, Harris J, Humphreys D, Malverus J, Gray NJ. “Be kind to young people so they feel at home”: a qualitative study of adolescents’ and service providers’ perceptions of youth-friendly sexual and reproductive health services in Vanuatu. BMC Health Serv Res. 2013;13(1): 455. 24176059 10.1186/1472-6963-13-455 PMC3842673

[41] Dahlbäck E, Maimbolwa M, Yamba CB, Kasonka L, Bergström S, Ransjö-Arvidson AB. Pregnancy loss: spontaneous and induced abortions among young women in Lusaka, Zambia. Cult Health Sex. 2010;12(3):247–62. 19904649 10.1080/13691050903353383

[42] Banerjee SK, Andersen K. Exploring the pathways of unsafe abortion in Madhya Pradesh, India. Glob Public Health. 2012;7(8):882–96. 22888792 10.1080/17441692.2012.702777

[43] Constant D, Grossman D, Lince N, Harries J. Self-induction of abortion among women accessing second-trimester abortion services in the public sector, Western Cape Province, South Africa: An exploratory study. S Afr Med J. 2014;104(4):302. 25118559 10.7196/samj.7408

[44] Singh S, Remez L, Sedgh G, Kwok L, Onda T. Abortion Worldwide 2017: Uneven Progress and Unequal Access. 2018 Mar 19 [cited 2024 Dec 7]; Available from: https://www.guttmacher.org/report/abortion-worldwide-2017

[45] Aniteye P, O’Brien B, Mayhew SH. Stigmatized by association: challenges for abortion service providers in Ghana. BMC Health Serv Res. 2016;16(1): 486. 27612453 10.1186/s12913-016-1733-7 PMC5018197

[46] Rehnström Loi U, Gemzell-Danielsson K, Faxelid E, Klingberg-Allvin M. Health care providers’ perceptions of and attitudes towards induced abortions in sub-Saharan Africa and Southeast Asia: a systematic literature review of qualitative and quantitative data. BMC Public Health. 2015;15(1): 139. 25886459 10.1186/s12889-015-1502-2 PMC4335425

[47] Miller ES, Grobman WA. Cost-effectiveness of transabdominal ultrasound for cervical length screening for preterm birth prevention. Am J Obstet Gynecol. 2013;209(6):e1-546. 10.1016/j.ajog.2013.08.013 23954533

[48] Faundes A. Misoprostol: Life-saving. Eur J Contracept Reprod Health Care. 2011;16(2):57–60. 21417559 10.3109/13625187.2011.561940

[49] Faúndes A, Santos LC, Carvalho M, Gras C. Post-abortion complications after interruption of pregnancy with misoprostol. Adv Contracept. 1996;12(1):1–9. 8739511 10.1007/BF01849540

[50] Rowlands S. Abortion pills: under whose control? J Fam Plann Reprod Health Care. 2012;38(2):117–22. 22454008 10.1136/jfprhc-2011-100232

[51] Allen R, O’Brien BM. Uses of misoprostol in obstetrics and gynecology. Rev Obstet Gynecol. 2009;2(3):159–68. 19826573 PMC2760893

[52] Prata N, Sreenivas A, Vahidnia F, Potts M. Saving maternal lives in resource-poor settings: Facing reality. Health Policy. 2009;89(2):131–48. 18620778 10.1016/j.healthpol.2008.05.007

[53] Sherris J, Bingham A, Burns MA, Girvin S, Westley E, Gomez PI. Misoprostol use in developing countries: results from a multicountry study. Int J Gynaecol Obstet. 2005;88(1):76–81. 15617717 10.1016/j.ijgo.2004.09.006

[54] Eschenbach DA. Treating spontaneous and induced septic abortions. Obstet Gynecol. 2015;125(5):1042–8. 25932831 10.1097/AOG.0000000000000795

[55] Corbett MR, Turner KL. Essential elements of postabortion care: origins, evolution and future directions. Int Fam Plan Perspect. 2003;29(3):106. 14519586 10.1363/ifpp.29.106.03

[56] Briozzo L, Vidiella G, Rodríguez F, Gorgoroso M, Faúndes A, Pons JE. A risk reduction strategy to prevent maternal deaths associated with unsafe abortion. Int J Gynaecol Obstet. 2006;95(2):221–6. 17010348 10.1016/j.ijgo.2006.07.013

[57] Briozzo L. From risk and harm reduction to decriminalizing abortion: The Uruguayan model for women’s rights. Intl J Gynecology & Obste [Internet]. 2016 Aug [cited 2025 Mar 11];134(S1). Available from: https://obgyn.onlinelibrary.wiley.com/doi/10.1016/j.ijgo.2016.06.003 10.1016/j.ijgo.2016.06.003 28748587

[58] Strong J. Men’s involvement in women’s abortion-related care: a scoping review of evidence from low- and middle-income countries. Sexual and Reproductive Health Matters. 2022;30(1):2040774. 35323104 10.1080/26410397.2022.2040774 PMC8956302

[59] Bennett LR. Single women’s experiences of premarital pregnancy and induced abortion in Lombok. Eastern Indonesia Reproductive Health Matters. 2001;9(17):37–43. 11468844 10.1016/s0968-8080(01)90006-0

[60] Gipson JD, Hindin MJ. “Having Another Child Would Be a Life or Death Situation for Her”: Understanding Pregnancy Termination Among Couples in Rural Bangladesh. Am J Public Health. 2008;98(10):1827–32. 18703439 10.2105/AJPH.2007.129262 PMC2636478

[61] Rehnström Loi U, Lindgren M, Faxelid E, Oguttu M, Klingberg-Allvin M. Decision-making preceding induced abortion: a qualitative study of women’s experiences in Kisumu, Kenya. Reprod Health. 2018;15(1): 166. 30285768 10.1186/s12978-018-0612-6 PMC6171301

[62] European Commission. Directorate General for International Cooperation and Development., University of St Andrews. Understanding gender inequality actions in the Pacific: ethnographic case studies and policy options. [Internet]. LU: Publications Office; 2016 [cited 2024 Dec 7]. Available from: https://data.europa.eu/doi/10.2841/896616

[63] Bankole A, Singh S, Haas T. Reasons why women have induced abortions: evidence from 27 countries. Int Fam Plan Perspect. 1998;24(3):117.

[64] Culwell KR, Hurwitz M. Addressing barriers to safe abortion. Intl J Gynecology & Obste [Internet]. 2013 May [cited 2024 Dec 7];121(S1). Available from: https://obgyn.onlinelibrary.wiley.com/doi/10.1016/j.ijgo.2013.02.003 10.1016/j.ijgo.2013.02.003 23477700

[65] Allotey P, Ravindran TKS, Sathivelu V. Trends in Abortion Policies in Low- and Middle-Income Countries. Annu Rev Public Health. 2021;42(1):505–18. 33138701 10.1146/annurev-publhealth-082619-102442

[66] Zhou J, Blaylock R, Harris M. Systematic review of early abortion services in low- and middle-income country primary care: potential for reverse innovation and application in the UK context. Glob Health. 2020;16(1): 91. 10.1186/s12992-020-00613-z PMC7524570 32993694

[67] Latt SM, Milner A, Kavanagh A. Abortion laws reform may reduce maternal mortality: an ecological study in 162 countries. BMC Womens Health. 2019;19(1):1. 30611257 10.1186/s12905-018-0705-y PMC6321671

[68] Committee on the Elimination of Discrimination against Women. Concluding observations on the sixth periodic report of Samoa. Geneva: United Nations; 2018. Report No.: CEDAW/C/WSM/CO/6.

[69] Committee on the Elimination of Discrimination against Women. Concluding observations on the combined initial to third periodic reports of Solomon Islands. Geneva: United Nations; 2014. Report No.: CEDAW/C/SLB/CO/1–3.

[70] Committee on the Elimination of Discrimination against Women. Concluding observations on the Fifth Periodic Report of Fiji. Geneva: United Nations; 2018. Report No.: CEDAW/C/FJI/CO/5.

[71] Committee on the Elimination of Discrimination against Women. Concluding observations of the Committee on the Elimination of Discrimination against Women: Papua New Guinea. Geneva: United Nations; 2010. Report No.: CEDAW/C/PNG/CO/3.

[72] Malhotra A, Nyblade L, Parasuraman S, MacQuarrie K, Kashyap N, Walia S. Realizing reproductive choice and rights abortion and contraception in India. Washington: International Center for Research on Women; 2003.

[73] Blystad A, Haukanes H, Tadele G, Haaland MES, Sambaiga R, Zulu JM, et al. The access paradox: abortion law, policy and practice in Ethiopia, Tanzania and Zambia. Int J Equity Health. 2019;18(1): 126. 31558147 10.1186/s12939-019-1024-0 PMC6764131

[74] Assifi AR, Berger B, Tunçalp Ö, Khosla R, Ganatra B. Women’s Awareness and Knowledge of Abortion Laws: A Systematic Review. Harper DM, editor. PLoS ONE. 2016;11(3):e0152224. 10.1371/journal.pone.0152224 PMC4807003 27010629

