# Supplementary material for: The state of abortion services in five Pacific Island countries: a legislative analysis and scoping review
Source: BMC Pregnancy Childbirth. 2025 Sep 30;25:970. doi: 10.1186/s12884-025-08005-0 (PMC12487553; doi:10.1186/s12884-025-08005-0)
Supplement: Supplementary file 1 — Supplementary Material 1 [file 12884_2025_8005_MOESM1_ESM.docx]

**Supplementary Table 1. Scoping review search strategy**

| **Database** | **Concept A – Abortion** | |
| --- | --- | --- |
| **Medline (OVID)** | Subject Headings (MeSH) | exp Abortion, Legal/ or exp Abortion, Therapeutic/ or Abortion, Criminal/ or misoprostol/ or Mifepristone/ or methotrexate/ or septic abortion/ or exp family planning services |
| **EMBASE** | Subject Headings (EMTREE) | septic abortion/ or hormonal abortion/ or incomplete abortion/ or mifepristone/ or mifepristone plus misoprostol/ or misoprostol/ or induced abortion/ or  abortion pump/ |
| **Medline/Embase** | Text words | (Abortion* or induc* abort* or unsafe* abortion* or post abortion care or post abortion servic* or incomplete abort* or dilatat* curettage* or misoprostol* or mifepristone* or methotrexate* or manual vacuum aspirat* or (terminat* adj3 pregnanc*) or (clandestin* adj3 abort*)).mp. |
| **Cinahl (EBSCO)** | CINAHL subject thesaurus | (MH "Abortion, Incomplete") OR (MH "Abortion, Induced+") OR (MH "Attitude to Abortion") OR (MH "Abortion, Criminal") OR (MH “Family Planning”) |
|  | Text words | Abortion* or unsafe abort* or post abort* care* or post abortion service* or incomplete abort* or dilatation and curettage* or vacuum aspiration* or misoprostol* or mifepristone* or methotrexate* termination of pregnancy or terminate pregnancy or clandestine abortion or illegal abortion or family planning |
| **Database** | **Concept B - Pacific Island countries** | |
| **Medline (OVID)** | Subject Headings (MeSH) | exp pacific islands/ or exp melanesia/ or exp micronesia/ |
| **Embase (OVID)** | Subject Headings (EMTREE) | exp pacific islands/ |
| **Medline/Embase** | Text-words | (American Samoa* or Fiji* or Papua New Guinea* or PNG* or Samoa* or Solomon Island* or Vanuatu* or (pacific* or melanesia* or micronesia* or polynesia*) or (Pago Pago* or Suva* or Port Moresby or Apia or Honiara or Port Vila*)).mp. |
| **Cinahl (EBSCO)** | CINAHL subject thesaurus | (MH “Pacific Islands”) |
|  | Text words | Pacific* or Melanesia* or Micronesia* or Polynesia* or American Samoa* or Fiji* or Papua New Guinea* or PNG* or Samoa* or Solomon Island* or Vanuatu* or Apia* or Pago Pago* or Port Moresby* or Honiara* or Suva* Port Vila* |
