# Supplementary material for: The state of abortion services in five Pacific Island countries: a legislative analysis and scoping review
Source: BMC Pregnancy Childbirth. 2025 Sep 30;25:970. doi: 10.1186/s12884-025-08005-0 (PMC12487553; doi:10.1186/s12884-025-08005-0)
Supplement: Supplementary file 2 — Supplementary Material 2 [file 12884_2025_8005_MOESM2_ESM.docx]

**Supplementary Table 2. Summary table of included papers**

| **Author** | **Year** | **Origin** | **Aim** | **Study-design** | **Method** | **Relevant key findings** |
| --- | --- | --- | --- | --- | --- | --- |
| Agyemang-Duah et al.^37^ | 2024 | PNG | To examine the association between intimate partner violence and pregnancy termination in PNG. | Quantitative | Analysis of secondary data from the PNG Demographic and Health Survey (2016-2018) | Overall, 6.3% of study participants (n=9,943) had ever terminated a pregnancy, and 61.5% reported having experienced intimate partner violence in the last 12 months preceding the survey.  Of those women who experienced IPV, 7.4% had ever terminated a pregnancy. Women who had experienced intimate partner violence had a 1.75 higher odds of reporting pregnancy termination (crude Odds Ratio: 1.75; 95% CI: 1.29–2.37) than women who did not experience intimate partner violence. |
| Asa et al.^27^ | 2012 | PNG | To determine  socio- demographic and other factors associated with illegal abortion in Eastern Highland Province. | Quantitative | Review of medical records | Abortion methods (n=27): misoprostol (85%), traditional herbal (7%) and mechanical means (7%).  Hospital stay length varied from 2-40 days. The most common method to manage post- abortion complications was evacuation of retained products of conception and antibiotics. |
| Babona et al.^34^ | 1974 | PNG | To analyse causes of maternal mortality in PNG between the years 1971 to 1972 | Quantitative | Analysis of reported maternal mortality data | There were four cases of maternal death due to “criminally induced” abortion out of the total recorded maternal deaths in PNG (n=364) between 1971 to 1972.gr |
| Burry et al.^36^ | 2023 | Pacific Island countries* | To understand the contexts and methods of illegal abortion, and how women and abortion are constructed in judges’ summing up, judgements, and sentencing. | Qualitative | Review of documents from court cases | Between the years 1960 to 2017, in the Pacific Islands examined, there were 18 illegal abortion cases. The individuals charged included seven medically trained abortion providers, six untrained abortion providers and six women seeking abortions.  Fear of others’ reactions to the pregnancy was a key motivator for women seeking abortions in many of the cases analysed. Women’s socio-economic circumstances were reasons for abortion as described in six cases. |
| Burry et al.^19^ | 2023 | Pacific Islands countries* | To understand how abortion is framed in media discourse in these Pacific Island countries. | Qualitative | Analysis of articles, opinion pieces and letters to the editor | Abortion was often positioned in opposition to gender ideology and national identity, with gender and national identity constructed by many commentators according to socially conservative, Christian doctrine. Abortion was also constructed as the killing of the “unborn,” Abortion was framed as often unsafe and a response to teenage pregnancy. |
| Bolnga et al.^29^ | 2021 | PNG | To investigate the hospital incidence, clinical management, and legal framework of self-induced abortions with misoprostol. | Quantitative | Semi-structured questionnaire | Reasons for abortion (n=51): unplanned pregnancy (57%) relationship problems (23%) medical issues (20%).  Women received assistance in acquiring misoprostol from (n=51): health workers (63%), friends (37%). |
| Bourdy et al.^32^ | 1992 | Vanuatu | To add to the knowledge regarding traditional medicine in Vanuatu used during the course of a woman’s reproductive life. | Qualitative | Interviews with community informants | A number of plants were described.  Chloroquine pills, lime guide and the juice of unripe papayas and alcohol are used.  D. gaudichaudianum and O. nutans are known among informants as fool proof abortive agents. |
| Kennedy et al.^40^ | 2013 | Vanuatu | To explore the barriers, enablers and SRH information and service delivery preferences of adolescents aged 15-19 years in Vanuatu.  To explore attitudes and perceptions of service providers and policymakers regarding the provision of SRH information and services to adolescents. | Qualitative | Focus group discussions  Semi-structured interviews | Traditional healers were identified as providers of SRH services, with some suggesting they were more affordable than clinic.  Some adolescents described accessing traditional healers for sexually transmitted infections or abortions. |
| Kopunye et al^39^ | 2021 | PNG | To conduct a study of PNG health professionals’ experience of induced abortion and their views on the provision of safe, accessible abortion services for  PNG women. | Quantitative | Questionnaires | Significant numbers of senior medical officers and nurses reported experience of women presenting following unsafe abortion. |
| Maviso et al.^38^ | 2024 | PNG | To estimate the prevalence and determine factors associated with pregnancy termination among married women aged 15-49 years in PNG. | Quantitative | Analysis of secondary data from the PNG Demographic and Health Survey (2016-2018) | Prevalence of pregnancy termination was 5.3% amongst married women (n=6,288). Nearly half of all pregnancy terminations occurred in the Highlands region.  Women aged 35-44 years, owned a mobile phone, and lived in urban areas were more likely to terminate a pregnancy. Women with unplanned pregnancies were 6.23 times more likely to terminate pregnancy.  Women who knew about modern contraceptive methods and made independent decisions for contraceptive use were 3.38 and 2.54 times more likely to terminate a pregnancy. |
| Ministry of Health and Medical Services et al. ^31^ | 2020 | Solomon Islands | To describe: the factors that contribute to unwanted pregnancy and abortion; the practices related to unwanted pregnancy and abortion; the health and other consequences of abortion; the supply and demand-side barriers that impact on timely access to quality post- abortion care; and the number of abortion complications presenting to health facilities and associated health systems costs of providing post- abortion care in the Solomon Islands. | Qualitative | Semi-structured interviews  Focus groups  Secondary data analysis of inpatient records at the National Referral Hospital. | In the absence of safe abortion services, women resort to a range of unsafe abortion methods including kastom (traditional) medicine, self- harm and use of misoprostol without clinical guidance or follow up.  Gaps exist in the quality of post-abortion care at public health facilities, such as provision of counselling and contraception to prevent repeat abortions. |
| Mitchell and Bennett^35^ | 2020 | Fiji | To understand the nature and impact of coercion being disclosed by women in the context of a wider piece of research into youth sexuality and sexual risk in Fiji. | Mixed methods | In-depth interviews  Participant observation | As described in a participant case study, a woman was pressured by her partner to have an abortion. Her partner made a doctor’s appointment for her at a private clinic. She ultimately yielded due to fear of being unable to raise the baby without her partner’s support. |
| USAID^26^ | 2011 | PNG | To assess unmet  needs of women for reproductive health, assess the uptake of clinical services and barriers to services. | Quantitative | Survey | Who conducted last  abortion (n=23): Friend, neighbour (48%), Self-inflicted (22%), Traditional healer (13%)  Abortion strategies (n= unknown): tablets (23%), traditional herbal  (45%), massage (9%), others (18%). |
| Sanga et al.^33^ | 2010 | PNG | To review information about women who died from pregnancy-related causes, both direct and indirect, in the Goroka General Hospital during the period 1st January 2005 to 31st May 2008. | Quantitative | Review of medical records | Of the participants’ medical records reviewed (n=21), puerperal sepsis and sepsis complicating unsafe abortion were the most common causes of maternal death accounting for 48% deaths. |
| Vallely et al.^28^ | 2015 | PNG | To describe from one setting in PNG, women’s reasons for resorting to unsafe abortion; the techniques they use; the consequences leading to hospital admission; and the reasons for abortion and seeking post- abortion care. | Mixed methods | Semi-structured interviews    In-depth interviews    Medical record reviews | Reasons for abortion include women’s desire to continue with their education, relationship problems and socio- cultural factors.  Misoprostol, physical and mechanical means, traditional herbs and spiritual beliefs were reported. |
| Vallely et al.^25^ | 2014 | PNG | To identify the types of complications that require hospital treatment as a result  of spontaneous and induced abortion and to explore the reasons why and under what circumstances women resort to induced abortion. | Mixed methods | Semi-structured interviews  Medical record reviews | Illegally obtained misoprostol was the most frequently used method.  Physical and mechanical means of abortion were widely reported. |
| Vanuatu Family Health Association.^30^ | 2014 | Vanuatu | To explore the attitudes and practices of communities with regards to abortions and unsafe abortions. | Mixed methods | Survey  Focus group discussions | Common methods used to induce abortion included: consuming lemon fruit, kastom (traditional) medicine, and vigorous exercise. Other methods included inserting objects into the uterus and taking contraceptive pills to cause a miscarriage.  Women’s reasons for abortion included: rape, incest, insufficient resources continuing career/school, too many children and fear of parents/others. |

*The Pacific Island countries examined by these studies include: Federated State of Micronesia, Fiji, Kiribati, Marshall Islands, Naura, Palau, PNG, Solomon Islands, Tonga, Tuvalu and Vanuatu. These studies have are eligible for inclusion on the basis that they cover the five Pacific Island countries focused on for this review.
